# Activation of Alpha Chymotrypsin by Three Phase Partitioning Is Accompanied by Aggregation

**DOI:** 10.1371/journal.pone.0049241

**Published:** 2012-12-11

**Authors:** Gulam Mohmad Rather, Joyeeta Mukherjee, Peter James Halling, Munishwar Nath Gupta

**Affiliations:** 1 Chemistry Department, Indian Institute of Technology Delhi, Hauz Khas, New Delhi, India; 2 Department of Pure and Applied Chemistry, University of Strathclyde, Scotland, United Kingdom; Aligarh Muslim University, India

## Abstract

Precipitation of alpha chymotrypsin in the simultaneous presence of ammonium sulphate and *t*-butanol (three phase partitioning) resulted in preparations which showed self aggregation of the enzyme molecules. Precipitation with increasing amounts of ammonium sulphate led to increasing size of aggregates. While light scattering estimated the hydrodynamic diameter of these aggregates in the range of 242–1124 nm; Nanoparticle tracking analysis (NTA) gave the value as 130–462 nm. Scanning electron microscopy and gel filtration on Sephadex G-200 showed extensive aggregation in these preparations. Transmission electron microscopy showed that the aggregates had irregular shapes. All the aggregates had about 3× higher catalytic activity than the native enzyme. These aggregates did not differ in λ_max_ of fluorescence emission which was around 340 nm. However, all the aggregates showed higher fluorescence emission intensity. Far-UV and near-UV circular dichroism also showed no significant structural changes as compared to the native molecule. Interestingly, HPLC gel filtration (on a hydroxylated silica column) gave 14 nm as the diameter for all preparations. Light scattering of preparations in the presence of 10% ethylene glycol also dissociated the aggregates to monomers of 14 nm. Both these results indicated that hydrophobic interactions were the driving force behind this aggregation. These results indicate: (1) Even without any major structural change, three phase partitioning led to protein molecules becoming highly prone to aggregation. (2) Different methods gave widely different estimates of sizes of aggregates. It was however possible to reconcile the data obtained with various approaches. (3) The nature of the gel filtration column is crucial and use of this technique for refolding and studying aggregation needs a rethink.

## Introduction

Three phase partitioning (TPP) of proteins is a simple process which was originally developed to concentrate proteins [1]. TPP essentially consists of precipitation of proteins as an interfacial layer when their aqueous solution is mixed with appropriate amounts of ammonium sulphate and *t*-butanol. The *t*-butanol rich layer and water layer separate out as two phases above and below the protein layer. The *t*-butanol, because of its shape and size does not penetrate the protein interior. However, it adheres to the protein and increases its buoyancy. That causes precipitated proteins to float rather than sink. It also reinforces the action of ammonium sulphate as precipitating agent. The two reagents obviously synergize as ammonium sulphate concentration used to cause precipitation of proteins during TPP is far lower than required for their salting-out during conventional precipitation by ammonium sulphate alone [2]. The interaction of the SO_4_^2−^ anion with proteins is more complex than is generally believed. As pointed out by Dennison and Lovrein (1997), the anion may operate in multiple ways: ionic strength effects, kosmotropy, cavity surface tension enhancement, osmotic stressor and exclusion-crowding chemical. In later years, TPP has become more widely used for protein purification [2]–[5] and protein refolding [6]. In some cases, it has been observed that subjecting the protein to TPP results in higher catalytic efficiency in both aqueous and low water media [7]–[11]. Our understanding of the structural changes brought about in a protein as a result of TPP treatment is largely based upon X-ray diffraction study of TPP treated Proteinase K [9]. TPP treatment of this protease led to a 3-fold increase in the caseinolytic activity of the enzyme. The comparison of X-ray diffraction (at 1.5 Å resolution) data between native Proteinase K and TPP-treated Proteinase K showed the following changes (a) While the H-bonding in the catalytic triad remained unchanged, water molecules in the substrate binding site were displaced. (b) Two acetate ions (TPP in that case was carried out in an acetate buffer) were present. One ion was in the active site and the other was located on the protein surface. (c) The structure in the case of TPP treated enzyme had a higher overall temperature factor [B = 19.7 Å^2^ as compared to 9.3 Å^2^ for the untreated enzyme]. Several amino acid side chains were found to exist in more than one conformation. Some of these side chains belonged to the active site. Overall, the enzyme molecule had a high flexibility which can lead to higher catalytic activity [12]. The increase in conformational flexibility also agrees with the observation that some TPP treated enzymes show higher catalytic activity in low water media (as compared to the untreated enzymes) [10], [11]. Enzymes in such media are known to acquire very rigid structures and that is largely responsible for their low activity [13].

Alpha chymotrypsin is another serine protease which has been extensively used in low water media for numerous applications such as synthesis of esters and peptides [14]. We had observed that TPP treatment of alpha chymotrypsin leads to a higher activity for the enzyme in both aqueous and low water media [10]. Unfortunately, our attempts at obtaining the structure of TPP treated alpha chymotrypsin by X-ray diffraction did not succeed [15]. During crystallization attempts, TPP treated alpha chymotrypsin underwent a selective autocatalytic cleavage near the N-terminus. The 14 amino acid long peptide turned out to have affinity for the active site of the enzyme. The X-ray diffraction data showed the peptide bound to the active site and the crystal was catalytically inactive [15]. Nevertheless, these observations also pointed out that TPP treated enzyme had a different conformation which was more vulnerable to autolysis during the long period of crystallization. The present study was undertaken to further understand the structural consequences of TPP treatment of alpha chymotrypsin. All studies on three phase partitioning of enzymes have focused on the precipitated protein obtained under optimum conditions (such as % saturation of ammonium sulphate) which results in maximum recovery of the activity [1], [2], [10]. Using alpha chymotrypsin as a model system, we also wanted to investigate what happens to structure and activity of the enzyme if a higher % saturation of ammonium sulphate is used. We found that TPP treatment of alpha chymotrypsin in fact leads to self aggregation of the enzyme. Considering that TPP has been reported as a useful refolding strategy [6], [16] and that such strategies generally work by inhibiting aggregation [17], these observations are surprising. These results may also be of wider interest in the context of our understanding of protein aggregation.

It was further found that different techniques generally used to look at aggregation like dynamic light scattering (DLS) [18], gel filtration [19], transmission electron microscopy (TEM) and the more recent nanoparticle tracking analysis (NTA) [18] all gave somewhat different quantitative pictures of this aggregation. This is known but underlines the fact that the frequently used practice of using a single technique to look at aggregation is likely to lead to misleading results. Our results also indicate that shape of the protein aggregate as a crucial parameter needs more attention in determining their size by various methods. Generally, many studies on protein aggregation do not include investigation of the shape (for example, by SEM and TEM). This practice though is quite common in the area of nanomaterials. The surprising finding was that the nature of gel filtration media was more crucial than hitherto believed. In asmuch as gel filtration is used both for detecting aggregates and as a refolding strategy [19], this observation may be useful to people working in these diverse areas.

## Results

While three phase partitioning has been used for purification of impure proteins [2]–[5], studies with pure enzymes have also been carried out and found often to result in increase in total units of activity [2], [9]–[11]. It has been shown in these earlier cases that this increase in enzyme activity is not due to removal of any contaminant protein but presumably results from some change(s) in the enzyme at the molecular level [2], [9], [10]. In the present studies, alpha chymotrypsin used was of the highest purity [65.6 U/mg protein using benzoyl tyrosine ethyl ester (BTEE) as a substrate (www.sigma.com, catalogue number 4126-1G) where 1 unit of this enzyme will hydrolyse 1 µmole of BTEE per min at pH, 7.8 at 25°C]. This is a purity level equal to or higher than that of alpha chymotrypsin preparations which have been used for structural work by us [10], [20] or by others [21], [22]. However, in order to further ensure that alpha chymotrypsin being used is pure, non reducing SDS-PAGE was run with two different amounts [20 µg and 60 µg] of protein. Single band was obtained in both cases [Supporting Information Fig. S1]. Even with high amount of 60 µg protein, no contaminating protein could be detected. The bands corresponded to a molecular weight of 25 kDa [23]. This is in agreement with the value of 25266 Da calculated from UniProt sequences [24].

**Figure 1:**
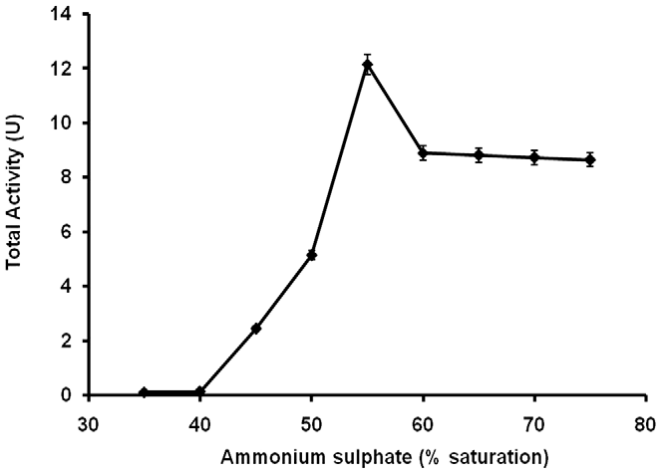
Activity curve of the alpha chymotrypsin using different ammonium sulphate concentration (w.v^−1^) in TPP. Alpha chymotrypsin: Units loaded = 4 U and Protein loaded = 3 mL, 3 mg mL^−1^, 0.02 M Sodium phosphate buffer, pH 7.8. Aqueous to *t*-butanol ratio: 1∶2 (v.v^−1^), Temperature: 25°C. Incubation time: 1 h. Activity assay: Caseinolytic. All the experiments were performed in triplicate and error bars represent the percentage error in each set of readings.

Fig. 1 shows the chymotrypsin activity (by caseinolytic assay) recovered in the interfacial layer after subjecting 4 U of alpha chymotrypsin to TPP. Only the total units of activity recovered from the interfacial layer of the precipitated protein is shown in this figure. These measurements were made after dissolving the precipitate and extensive dialysis. One unit of caseinolytic activity is defined as the amount of enzyme that released trichloroacetic acid (TCA) soluble peptides equivalent to 1 µmole L-tyrosine per minute under assay conditions [25]. About 3 fold increase in total activity recovered when 55% saturation of ammonium sulphate was used during TPP is in agreement with the results reported earlier for alpha chymotrypsin [10]. Similar increases in catalytic activity of other enzymes after TPP treatment have been reported by us [9], [11] and by others [7], [8]. What was interesting to observe was that even with higher % saturation of ammonium sulphate, the total activity recovered remained well above 100% (i.e. 4 U). The enzyme obtained in interfacial layers at ‘x’ % saturation of ammonium sulphate is called ‘x% IP’ during subsequent discussion.

**Figure 2:**
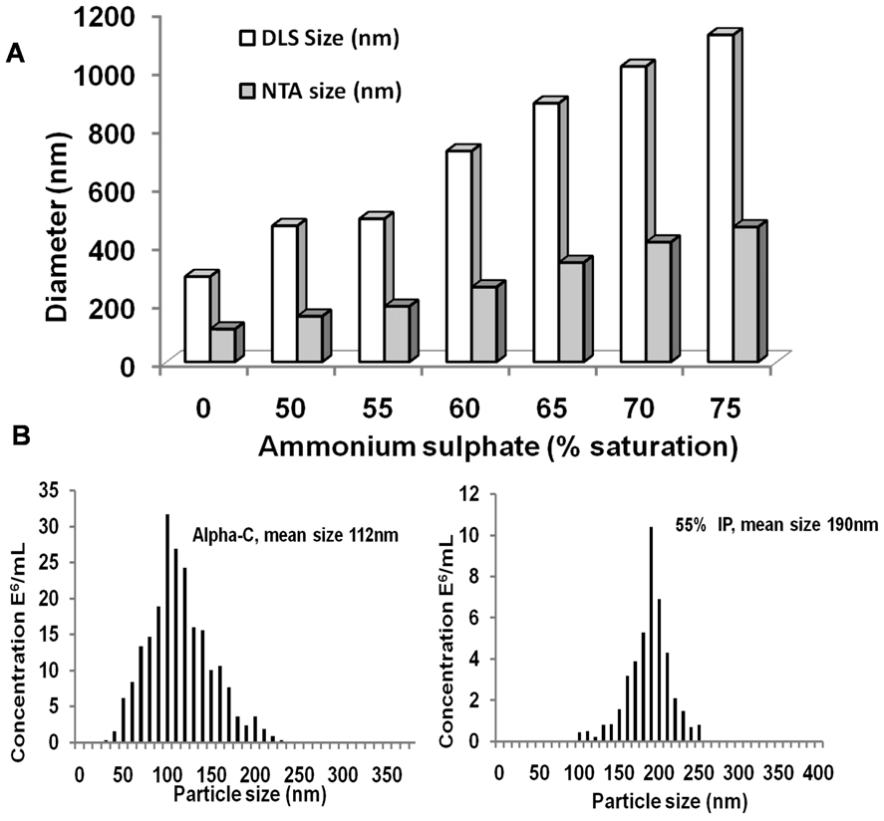
TPP treated alpha chymotrypsin preparations. (A) Comparison of sizes by Nanoparticle tracking analysis and DLS (B) Size distribution by Nanoparticle tracking analysis. The sizes are number mean diameters (NTA data) and intensity mean diameters (DLS data). Where a suspension is not monodisperse, the intensity mean will always be larger than number mean.

The hydrodynamic diameters of various preparations when dissolved in distilled water were determined by light scattering (Table 1, Fig. 2A) and NTA, (Fig. 2A), respectively. The latter carries out nanoparticle tracking analysis (NTA) of particles in Brownian motion. Although particles are too small to image, they can be tracked as scattering centres, and the mean amplitudes of their motion reflect their size. Unlike DLS or photo correlation spectroscopy, the particle size distribution obtained by NTA is direct number/frequency distribution. As NTA looks at number/frequency distribution, these measurements do not ignore particles of small sizes. The NTA, however, has lower limit of detection of about 25–35 nm [26]. DLS tends to miss smaller particles in the presence of larger ones – hence under these conditions NTA will give a smaller mean, because it still detects the smaller particles [26]. It was decided to employ this technique as well for looking at the size of specie(s) in TPP treated alpha chymotrypsin. Both techniques showed that TPP treatment resulted in aggregation of alpha chymotrypsin.

**Table 1:** Hydrodynamic diameter of alpha chymotrypsin preparations measured by Light Scattering (DLS).

Enzyme Preparation	Water	Buffer		#Buffer+1 M NaCl
		A*	B**	
Alpha-C	288	14	16	18
40% IP	244	242	250	680
50% IP	466	462	496	730
55% IP	490	492	490	752
60% IP	722	726	744	768
65% IP	886	888	1166	926
70% IP	1012	1006	1250	1022
80% IP	1130	1124	1284	1142

Buffer used was 0.02 M sodium phosphate, pH 7.8. All the hydrodynamic diameter values are in nm. All size measurements were carried out in triplicates and the % error between each set of readings was less than 2%.

* The values in Column A were obtained by measurements with samples after filtration through 0.22 µm filter.

** The values in Column B results from the corresponding measurements carried out directly with samples, that is, without subjecting the samples through any filtration.

# The values in this column were obtained after filtration through 0.22 µm filter.

**Figure 3:**
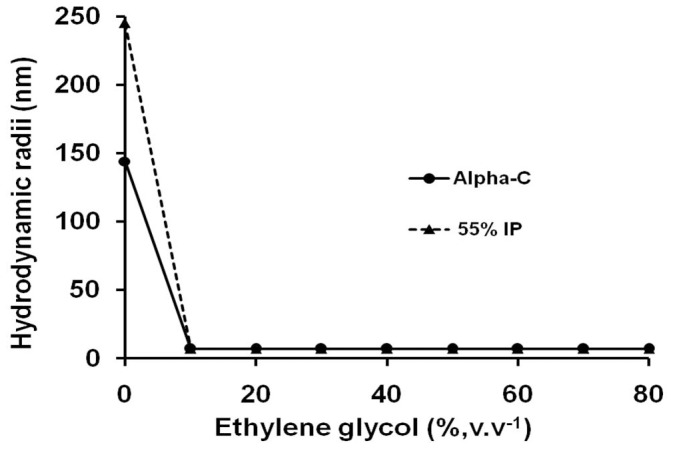
Effect of ethylene glycol on the size of the alpha chymotrypsin preparations (prepared in distilled water). All the experiments were performed in duplicates and the percentage error in each set of readings is less than 3%.

Fig. 2B shows illustrative examples of particle size vs concentration plots obtained from NTA with two preparations (Supporting Information Fig. S2, Fig. S3 containing the videos of Brownian motion). While both DLS and NTA showed enhanced aggregation among the TPP treated preparations, expectedly (see earlier for the basic differences in the principle) NTA gave much smaller sizes for various aggregates (Fig. 2A). The Brownian motion of the aggregates can be seen in the video (Supporting Information Fig. S3–Fig. S5). The Brownian motion can be seen to become slower in the sequence, alpha chymotrypsin >55% IP >65% IP >75% IP. The particle size distribution in both alpha chymotrypsin and TPP treated alpha chymotrypsin (with 55% saturation of ammonium sulphate) showed that there was considerable polydispersity. The particle size obtained by both methods showed the following features (a) Even alpha chymotrypsin was present as aggregates in distilled water (Table 1, Fig. 2A). The tendency of alpha chymotrypsin to aggregate is known. It has been shown that the size of alpha chymotrypsin obtained by the light scattering method was highly dependent upon the ionic strength of the buffer [27]. Light scattering and NTA gave the hydrodynamic diameter and particle size as 282 nm and 112 nm, respectively, (Table 1, Fig. 2A). It should be emphasized that in buffered solutions (wherein the size measurements are normally made), untreated alpha chymotrypsin gave size of ∼14 nm which is in agreement with the reported hydrodynamic diameter of the alpha chymotrypsin molecule [28]. This confirmed that no artifacts were being observed. (b) Increase in % saturation of ammonium sulphate used during TPP led to an increase in the size of the aggregates (Table 1, Fig. 2A). Adding either ammonium sulphate or *t*-butanol alone (at the same concentration as during TPP) did not result in the increase in size of alpha chymotrypsin (Neither chemical when added alone causes precipitation of the protein nor these leads to phase separation. So these measurements were made directly with the solution). Light scattering of these control preparations gave hydrodynamic diameter of 10–14 nm (data not shown). That is in agreement with the reported hydrodynamic diameter of 6–14 nm of alpha chymotrypsin by light scattering [28]. Hence aggregation of alpha chymotrypsin molecules did not take place when either ammonium sulphate or *t*-butanol alone were present but resulted only from TPP treatment. These measurements with TPP treated samples were made in distilled water. The interfacial layers were dissolved in 0.02 M sodium phosphate buffer, pH 7.8 and dialysed extensively against distilled water (column heading “water”, Table 1). The hydrodynamic diameters of the various preparations were also measured in solutions of different ionic strength by DLS (column heading “buffer”, Table 1). In the presence of moderate ionic strength (0.02 M sodium phosphate buffer, pH 7.8) alpha chymotrypsin ceased to show any aggregation behavior. However, TPP subjected preparations were still in the form of aggregates (Table 1). It was found that the buffer with higher ionic strength (i.e. containing 1 M NaCl) actually promoted further aggregation. This effect was most pronounced for TPP preparations obtained with % saturation of ammonium sulphate in the range of 40%–55%. Aggregation mediated by hydrophobic interactions is known to be salt promoted. This effect follows the Hofmeister series. So, while the most efficient salts are sodium sulphate and ammonium sulphate, even “weaker” salts like sodium chloride can promote this effect [29]. The aggregate (obtained with 55% saturation of ammonium sulphate and dialyzed against distilled water) was indeed partially dissociated in the presence of ≥10% (v/v) concentration of ethylene glycol (Fig. 3) which is known to decrease hydrophobic interactions [30]. Hence, TPP treatment led to alpha chymotrypsin becoming more prone to aggregation and this aggregation was mediated by hydrophobic interactions.

The increase in size of aggregates when % saturation of ammonium sulphate is increased points towards salt promoted aggregation. That is understandable since this aggregate formation seems to be mediated by hydrophobic interactions.

**Figure 4:**
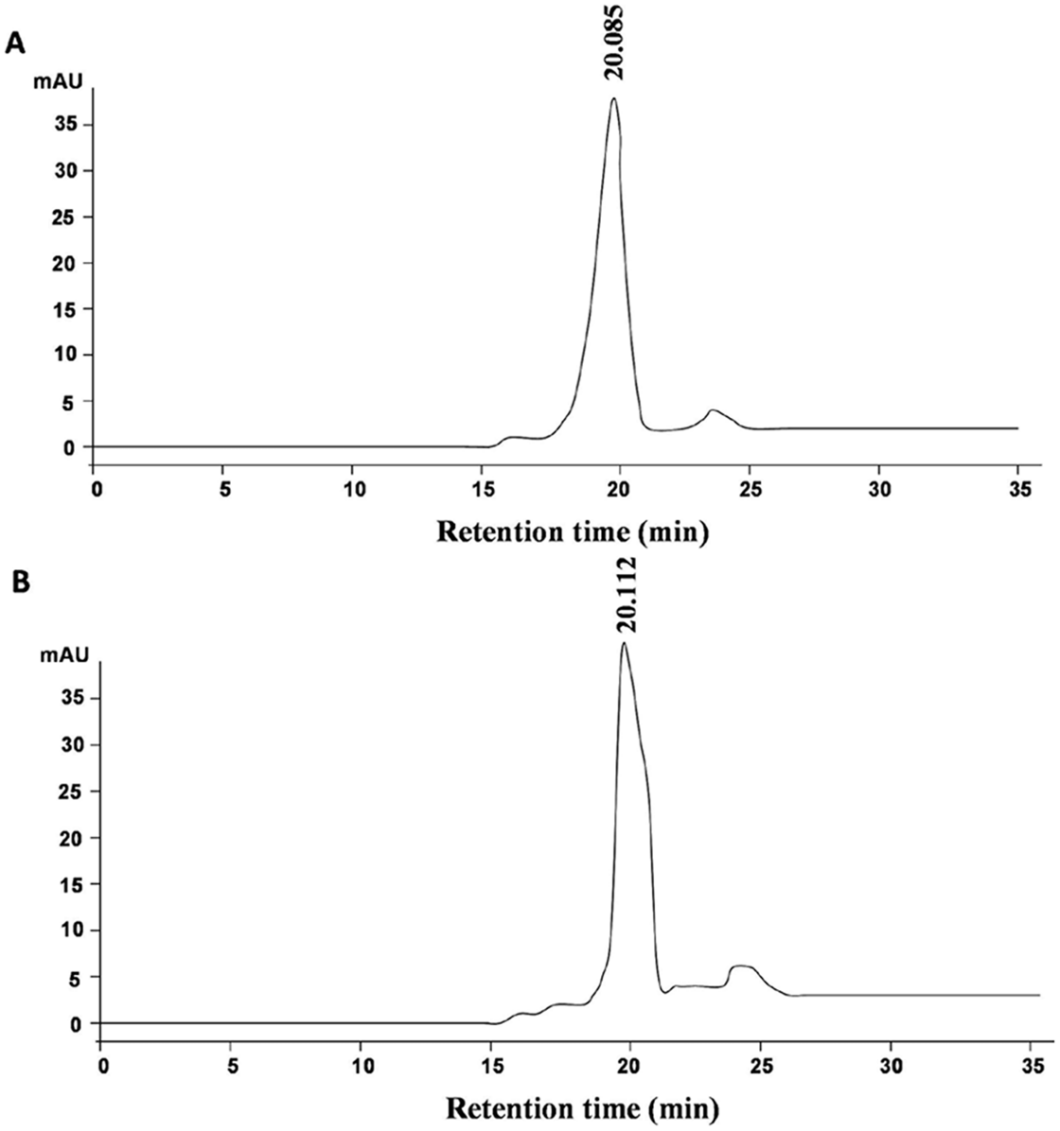
HPLC Gel filtration chromatograms of: (A) Native alpha chymotrypsin solution (B) TPP treated alpha chymotrypsin with 55% saturation of ammonium sulphate.

**Figure 5:**
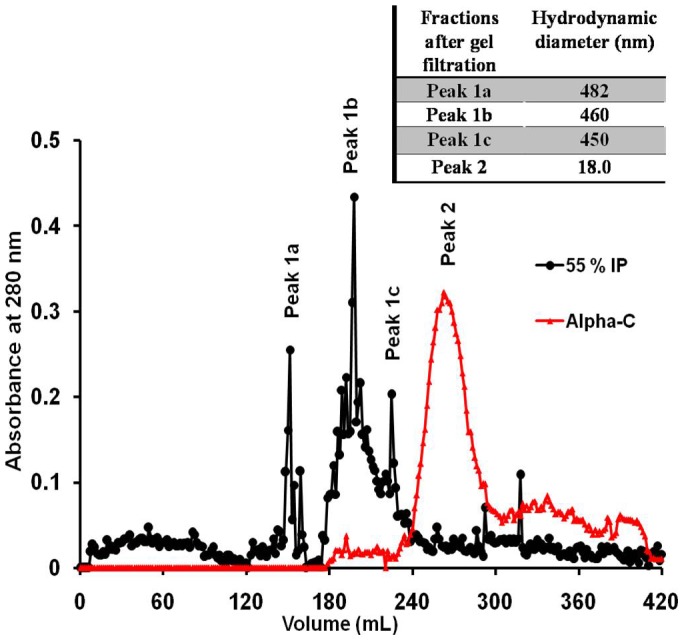
Gel filtration chromatogram of Alpha-C and 55% IP, separately on a Sephadex G-200 column. Inlay table shows the DLS size of the major peaks of chromatogram of 55% IP sample.

In order to look at the sizes of these aggregates by yet another technique, HPLC gel filtration was employed, but surprisingly both alpha chymotrypsin and TPP treated alpha chymotrypsin (with 55% saturation of ammonium sulphate) showed similar sizes in solutions in 0.02 M sodium phosphate buffer, pH 7.8 (Fig. 4A and 4B). It was thought that perhaps the nature of the HPLC gel filtration column (hydroxylated silica) has played a role in dissociating the aggregates. This suspicion was based upon the observation that this aggregation was mediated by hydrophobic interaction and even a limited hydrophobicity of the HPLC gel filtration column (with multiple theoretical plates) may have led to dissociation of these aggregates. Hence a gel filtration column packed with more hydrophilic beads, Sephadex G-200 was used. In this case distinct separate peaks were obtained for alpha chymotrypsin and TPP treated alpha chymotrypsin (with 55% saturation of ammonium sulphate) in the same buffer (0.02 M sodium phosphate buffer, pH 7.8). All the components of the TPP treated preparation eluted out earlier than alpha chymotrypsin, indicating that they existed as aggregates (Fig. 5). The TPP treated alpha chymotrypsin gave multiple peaks on gel filtration which is indicative of the fact that the aggregates are not of uniform size (that is in agreement with frequency distribution provided by NTA (Fig. 2B). The fractions corresponding to the different peaks were collected and their sizes were determined using light scattering and they were found to have hydrodynamic diameters in the range of 450–482 nm which agrees well with what was found for this TPP preparation before (492 nm) (inlay of Fig. 5). To confirm the fact that HPLC gel filtration does give different results, these fractions (obtained from gel filtration of this TPP treated preparation on the Sephadex-G200 column) were injected into the HPLC column. All the fractions appeared at the same retention time as the native alpha chymotrypsin (Supporting information Fig. S6). The molecular weight markers run on the Sephadex gel filtration column gave us a rough estimate of the molecular weights of the aggregates (Supporting information Fig. S7). The SEM images show that TPP treatment did result in considerable aggregation of the enzyme (Supporting Information Fig. S8). The TEM images further showed that the aggregates were highly irregular in shape (Supporting Information Fig. S9). Hence TPP treatment led to aggregation of alpha chymotrypsin and results obtained with different techniques agreed in general that these TPP treated preparations were in fact aggregated forms of the enzyme, though more enzymatically active (as measured by caseinolytic activity).

**Figure 6:**
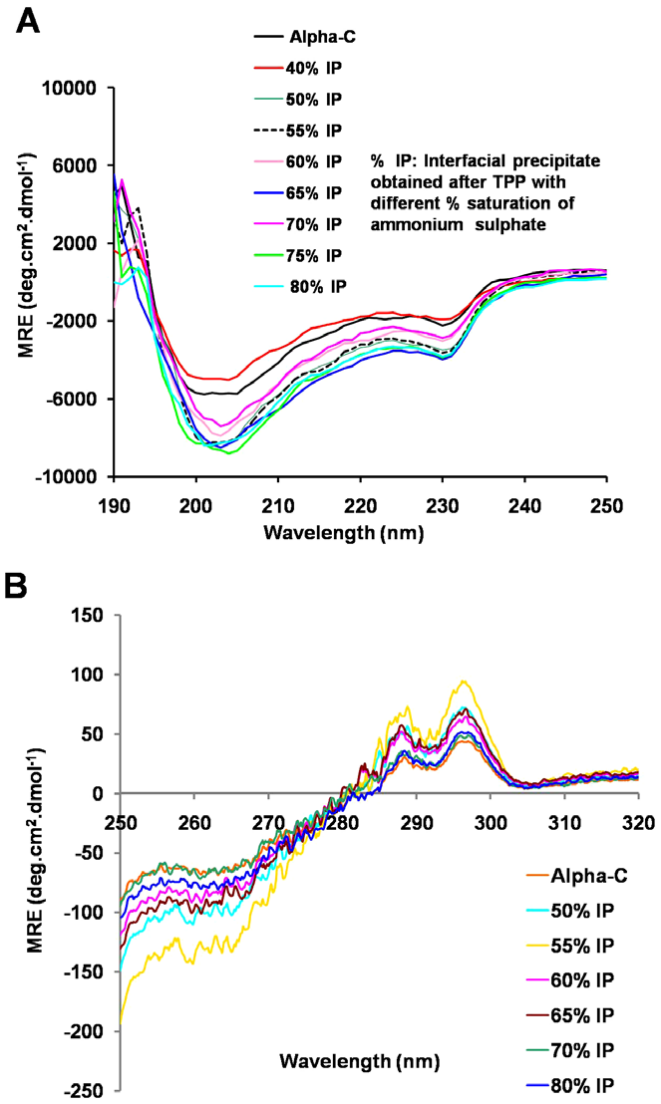
Circular Dichroism spectroscopy of various preparations of TPP treated alpha chymotrypsin. (A) Far-UV CD spectra (B) Near-UV CD spectra.

Fig. 6A shows the far UV CD spectra of these various TPP treated preparations obtained with various % saturation of ammonium sulphate. The spectrum shows a negative peak at 230 nm and 202 nm. The far-UV CD spectrum of alpha chymotrypsin is reported to be characterized by negative peaks at 229 nm and 202 nm [21], [31]. “Alpha chymotrypsin is a type of all-β protein characterized by a CD spectrum, which resembles that of random coil conformation. Crystal structure data showed that this kind of protein consists of anti-parallel pleated β-sheets which are either highly distorted or form very short irregular strands. This may cause the negative CD band to shift from the ideal β-sheet position (210–220 nm) towards the 200 nm region” [21]. Hence, according to the earlier workers, the CD spectrum is characterized by the negative band around 230 nm and a global minimum around 200 nm. Table 2 summarizes the secondary structure contents calculated from the above far UV CD spectra. Our results (8% alpha helix and 35% beta sheet for native alpha chymotrypsin, Table 2) are very similar to the reported values [20], [21], [32]. It is interesting to note that the TPP treated preparations showed no significant changes in α-helix or β-sheet structures as compared to the native structure of alpha chymotrypsin. The near UV CD spectra of both untreated and TPP treated alpha chymotrypsin showed positive maxima at 296 nm and 287 nm and a negative peak at around 260 nm (Fig. 6B) which is in agreement with the characteristic spectra of alpha chymotrypsin [21], [31]. The near UV CD spectra (Fig. 6B) reflect the tertiary structure (in terms of orientations of aromatic amino acids) [33]. These results show that secondary and tertiary structures of the molecules in these aggregates have not changed in any significant way. This correlates well with the fact that aggregation did not result in any loss of activity.

**Table 2:** Secondary structure content (%) as determined by K2D2 software from the Far-UV CD spectra as given in Figure 6A.

Enzyme Preparation	α-helix	β-sheet	Random coil
Alpha-C	8	35	57
40% IP	9	44	47
50% IP	8	41	51
55% IP	8	41	51
60% IP	8	41	51
65% IP	9	35	56
70% IP	9	35	56
75% IP	9	35	56
80% IP	9	35	56

**Figure 7:**
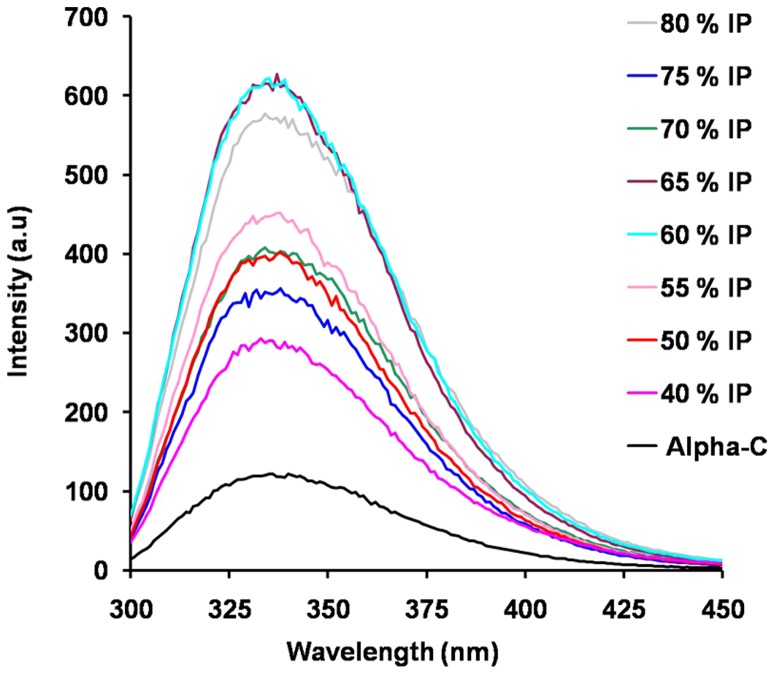
Fluorescence emission spectra of TPP treated alpha chymotrypsin preparations. The emission spectrum was measured using excitation at 295 nm. The excitation and emission slit widths were kept at 5 nm.

Alpha chymotrypsin has been extensively investigated using fluorescence emission spectroscopy. The molecule has 8 Trp residues which are excited at 295 nm [34]. Fig. 7 shows the fluorescence emission spectra originating from Trp residues of alpha chymotrypsin in these various TPP treated preparations. While there was no shift in λ_max_ of emission spectra, all TPP treated preparations showed higher fluorescence intensity than untreated alpha chymotrypsin. The fluorescence emission spectrum tracks the polarity of the micro environment of Trp residues and their specific interactions with neighbouring side chains. The increase in fluorescence intensity reflects either Trp residues getting buried or the abolition of some quenching interactions with neighbouring side chains. The absence of any red shift however rules out any significant unfolding of the molecules present in the aggregates [35], [36]. The latter is in agreement with the absence of any significant changes in the near-UV CD spectra [Fig. 6B]. The surface hydrophobicity of the aggregates showed decrease in value [Table 3; Supporting Information Fig. S10].

**Table 3:** Surface hydrophobicity measurement of alpha chymotrypsin preparations by ANS titration using Fluorescence Spectroscopy.

Enzyme Preparation	So (µM)^−1^
Alpha-C	1.242
50% IP	0.786
55% IP	1.168
80% IP	0.895

Each experiment of surface hydrophobicity measurements using ANS was carried out in duplicates and the error between the two set of readings was less than 3%.

## Discussion

To sum up the main results, we observed the following: TPP treatment of alpha chymotrypsin leads to higher caseinolytic (proteolytic) activity for the enzyme. There is a minimum % saturation of ammonium sulphate which is required for precipitation of alpha chymotrypsin in the interfacial layer. Beyond this % saturation of ammonium sulphate, all alpha chymotrypsin preparations after TPP treatment showed this increase in total amount of activity. This increase in total amount of activity upon TPP treatment of (even) a pure enzyme has been well known [2], [9]–[11]. However, the earlier studies were limited to carrying out TPP at one single optimum % saturation of ammonium sulphate. From the results of the current work, it is seen that even if % saturation of ammonium sulphate is increased, the proteolytic activity did not go back to the activity shown by untreated alpha chymotrypsin. Alpha chymotrypsin upon TPP treatment (as described above) undergoes aggregation. This aggregation seems to be mediated by hydrophobic interaction. These aggregates were of highly irregular shapes as seen by TEM. Intrinsic fluorescence emission spectra of these aggregates show that at least some of the 8 Trp residues of alpha chymotrypsin are now less accessible to the solvent. However, absence of red shift indicates that there has been no collapse of tertiary structure [35]. Far-UV CD spectra showed no significant changes in the secondary structure of alpha chymotrypsin. Near-UV CD spectra confirmed fluorescence results and showed absence of any significant changes in tertiary structure of alpha chymotrypsin in all these aggregates. All techniques (DLS, NTA, HPLC gel filtration, gel filtration on Sephadex-G200, SEM, TEM) show that TPP treatment led to aggregation of alpha chymotrypsin.

DLS and NTA data on detection of aggregates inspite of prefiltration through a 0.22 µm filter needs some comments. It is noteworthy that the average size of aggregates detected by DLS was only marginally bigger when this prefiltration step was omitted (Table 1). Equally important, after passing 55% IP through 0.22 µm filter, the amount of protein decreased by about 25%. This was based upon absorbance at 280 nm (data not shown). The indicated cut-off of 0.22 µm actually means a range of pore sizes in the membrane and hence a percentage of particles slightly bigger than 0.22 µm are expected to go through. However, similar average size seen by DLS for both filtered and unfiltered samples indicate that some other mechanism is operating. Both nucleation-elongation and isodesmic mechanisms of aggregation are known [37]. Both are compatible with the notion that smaller aggregates are in equilibrium with bigger aggregates in TPP treated samples. After bigger aggregates are removed, smaller aggregates again associate to form bigger aggregates. The somewhat larger differences between sizes of filtered and unfiltered aggregates (Column A and B in Table 1) when TPP was carried out at higher % saturation of ammonium sulphate does not contradict this interpretation. As the size of aggregates increase, less may pass through 0.22 µm filter.

The erratic elution pattern of the soluble aggregates on sephadex G-200 is presumably due to highly irregular shapes of the aggregates. This behavior of specie of such odd shapes is expected to be different from the one shown by globular proteins of more regular shapes. These soluble aggregates constitute a polydispersed preparation wherein the aggregates of various sizes are presumably in equilibria. The single peak shown by all TPP treated proteins at the position of monomeric alpha chymotrypsin on the HPLC gel filtration should be of interest to other workers who use such columns for refolding or studying aggregation.

Fluorescence emission spectra of the aggregates do differ from native alpha chymotrypsin in the present case. These do not indicate any drastic change like red shift which has been observed when insoluble aggregates were formed by chemical denaturation [35].

Surface hydrophobicity of the aggregates showed decrease in value (Table 3). This agrees with the model that “native like” monomers of alpha chymotrypsin interacted via hydrophobic surfaces leading to overall decrease in surface hydrophobicity. Absence of any trend in surface hydrophobicity presumably originates in the irregular shapes of these aggregates. A monomer binding to a preexisting aggregate would interact depending upon the morphology (hence surface) of the preexisting aggregate.

What could be the reasons for TPP treated alpha chymotrypsin to form aggregates? Protein aggregation was believed to be caused by denatured/misfolded protein molecules combining together via exposed hydrophobic patches. In recent years, this notion has undergone drastic change. For example, it has been reported that inclusion bodies of recombinant proteins expressed in bacterial hosts are not necessarily 100% inactive but may contain aggregates of active molecules [38], [39]. In the context of amyloid formation (being extensively investigated because of their relevance to neurodegenerative diseases), Marcon et al., (2006) [40] mentions that “some proteins such as Ure2p and lithostatin, are found to aggregate under non-destabilizing physiological conditions and formed fibrils in which the individual protein molecules retained a native like structure”. Interestingly enough, later in the same article, while discussing conditions that promote aggregation, they write “to choose solution conditions that destabilize the native state (i.e., that facilitate its flexibility, favoring the population of quasi-native states, or reduce the free energy of unfolding)”. The general picture which has emerged is that even “native like” structures can undergo aggregation. It has been observed that even subtle conformational changes can cause aggregation [41].

From the results obtained in the present work, it appears that TPP treatment leads to some very minor changes in the structure of alpha chymotrypsin. These native like structures aggregate. The enzymatic activity of alpha chymotrypsin is not merely retained in the aggregates but actually enhanced. This is presumably because, like proteinase-K, the monomeric alpha chymotrypsin after TPP treatment has a more flexible structure. This structure leads to aggregation and is retained in the aggregate. The higher activity shown by TPP treated alpha chymotrypsin [10] and many other TPP treated proteins [11] in low water containing organic solvents is a strong indication of more flexible molecules. The bound water layer of the enzymes in such media is stripped off and extensive intramolecular weak interactions make the enzyme molecules highly rigid and relatively less active. Any treatment which leads to a more flexible molecule is known to enhance enzyme activity in such solvents [13].

Finally, it may be interesting to correlate some other current understanding of protein aggregation with our results. Freiden, 2007 [37] has pointed out that intrinsically disordered proteins (also called “Natively unfolded”) have one feature: these have “ensembles of states” in which side chain positions differ from fixed positions. This is very reminiscent of what was observed in the case of X-ray diffraction of TPP treated proteinase K [9]. IDPs (intrinsically disordered proteins) have other properties as well and are known to aggregate to form amyloid aggregates. Hence, it is not our case that TPP treatment leads to an IDP like structure for alpha chymotrypsin. It is nevertheless interesting that TPP treatment is known to lead to side chains acquiring flexible conformations and in alpha chymotrypsin it led to aggregation. It could be because alpha chymotrypsin to start with is known to have a rather high percentage of hydrophobic surfaces and is quite prone to aggregation. A very small change in its structure by TPP treatment “tipped” the balance and the protein aggregated. Hence, TPP treated alpha chymotrypsin and its properties may be of interest in a wider context.

## Materials and Methods

### Materials

Alpha chymotrypsin was purchased from Sigma Chemical Co., St. Louis, MO, USA. *t*-butanol and ethylene glycol were purchased from Merck Pvt. Ltd, Mumbai India. All other chemicals and reagents used were of analytical grade.

### Polyacrylamide gel electrophoresis

SDS-PAGE of the alpha chymotrypsin samples using 15% gel was performed according to Hames (1986) [23], using a Genei gel electrophoresis unit (Bangalore Genei, Bangalore, India). The protein bands on the gel were visualized using Coomassie Blue stain. SDS-PAGE was performed under non-reducing conditions [23].

### Three phase partitioning of alpha chymotrypsin

Alpha chymotrypsin (3 mL, 3 mg.mL^−1^ in 0.02 M sodium phosphate buffer, pH 7.8) was saturated with different % saturation of ammonium sulfate. This was followed by the addition of 6 mL of *t*-butanol; the solution was vortexed and allowed to stand at 25°C for one hour. The solution was then centrifuged (2000 g, 10 min). The lower aqueous and upper organic layers were separated using a pasteur pipette. The interfacial precipitate was dissolved in 1 mL of 0.02 M sodium phosphate buffer, pH 7.8 and the samples were dialyzed against the same buffer (2 litres) at 4°C with changes after every two hours for the first 12 hours and a final change after 24 hours; the dissolved precipitate was then used for further studies. The untreated enzyme, which was incubated in 0.02 M sodium phosphate buffer, pH 7.8 for the same period as the TPP- treated enzyme, then dialyzed against the same buffer was regarded as the control (Alpha-C) and its activity was taken as 100% for all subsequent activity measurements.

### Enzyme assay and protein estimation

The caseinolytic activity was estimated by determining the amount of trichloroacetic acid (TCA) - soluble peptides [42] and the protein was estimated by measuring the absorbance at 280 nm assuming a molar extinction coefficient of _280_E^1%^ = 20.4 [43].

### Circular dichroism measurements

The far-UV and near-UV circular dichroism (CD) spectrum of various species of TPP-treated alpha chymotrypsin were recorded with a spectropolarimeter (JASCO 815) with protein samples at a concentration of 0.2 mg.mL^−1^ (as measured by absorbance at 280 nm) in 0.02 M sodium phosphate buffer, pH 7.8 at 25°C. The spectrum was recorded using a 2 nm band width, 8 s response rate, scan rate of 50 nm.min^−1^ and a 0.1 nm pitch for far UV. For near UV, protein samples at a concentration of 1.5 mg.mL^−1^ (as measured by absorbance at 280 nm) in 0.02 M sodium phosphate buffer, pH 7.8 at 25°C were used in a 0.1 cm path length cell. The spectrum was recorded using 1 nm band width, 1 s response rate, scan rate of 20 nm.min^−1^ and a 0.2 nm data pitch. Four accumulations were recorded and averaged. The blank spectrum of an aqueous buffer was used to correct the observed spectrum of the sample. The CD data were expressed as mean residual ellipticity in deg.cm^2^.dmol^−1^. The spectrum was subjected to secondary structure analysis using k2d2 online software.

### Fluorescence spectroscopy and surface hydrophobicity index (So) measurements

Fluorescence spectra were recorded at 25°C with a Varian Cary Eclipse, fluorescence spectrophotometer using a 1 cm cuvette. The emission spectrum was measured using excitation at 295 nm. The excitation and emission slit widths were kept at 5 nm. All fluorescence spectra were normalized and corrected for buffer contributions.

Surface hydrophobicity parameter for the different preparations of TPP-treated alpha chymotrypsin was measured by a fluorimetric approach, applying the slope method of Kato and Nakai [44]. An excess of fluorescent probe ANS at 100 µM was titrated by a typical protein concentration range of 0–7.0 µM (as measured by absorbance at 280 nm) in 0.02 M sodium phosphate buffer, pH 7.0. Excitation and emission wavelengths were 390 and 470 nm respectively. Relative fluorescent intensity (FR) calculated as [(F-F_o_)/F_o_]×10 was plotted as a function of protein concentration [45] and the slope of the regression line was taken as the So value.

### Light scattering measurements

Dynamic light scattering (DLS) measurements were performed at 25°C using a RiNA GmbH laser spectroscatter 201 (Berlin, Germany). The hydrodynamic radii (Rh) were calculated by software PMgr v3.01p17 supplied with the instrument. The protein solutions of TPP-treated alpha chymotrypsin were centrifuged at 8000 g for 20 min and all protein solutions were filtered using 0.22-µm pore size cut-off filters. The sample solution (100 µL) was carefully transferred to a cylindrical cuvette (1.5 mm path length), taking care to avoid bubble formation in the solution. A series of measurements with a sampling time of 30 s and a wait time of 1 s was conducted. A diode laser of wavelength 680 nm was used as the source. The scattered light was collected at a fixed angle of 90° by a lens system and directed through a glass fibre to a photomultiplier. Its output pulses were processed by the autocorrelator.

#### Nanoparticle tracking analysis

Light scattering measurements were performed at 25°C using a NanoSight LM10 (Wiltshire, UK). The solutions of TPP-treated alpha chymotrypsin were filtered using 0.22-µm pore size cut-off filters and 0.3 mL sample was injected into the viewing chamber with sterile syringes. The image analysis was done using the Nanoparticle Tracking Analysis (NTA 2.0) software. The samples were measured for 10 s with the optimized camera settings.

### Effect of Ethylene Glycol on alpha chymotrypsin aggregates

Different preparations of TPP-treated alpha chymotrypsin dissolved in distilled water were incubated with different concentrations of ethylene glycol for 30 min at 25°C and then the sizes were measured by light scattering.

### Molecular weight determination by HPLC Gel filtration

HPLC analysis of different preparations of TPP-treated alpha chymotrypsin was obtained on the Agilent 1100 HPLC system equipped with a gel filtration column (TSK G2000SWXL column, TOSOH bioscience, Japan), prequilibrated with 0.1 M sodium phosphate buffer, pH 7.8 containing 0.1 M sodium sulphate. The column was calibrated with standard molecular weight markers. The peaks were analyzed using a UV detector at 280 nm.

### Gel filtration chromatography

The TPP treated alpha chymotrypsin preparation with 55% saturation of ammonium sulphate was loaded on a Sephadex G-200 column (70 cm×2.5 cm; 340 mL bed volume) pre-equilibrated with two bed volumes of 0.02 M sodium phosphate buffer (pH 7.8). Fractions (1.5 mL) were collected and read at 280 nm. The native alpha chymotrypsin was also loaded on the same column after two-three bed volume washings and eluted with the same buffer.

Similarly thyroglobulin, beta-amylase, bovine serum albumin and alpha chymotrypsin (as gel filtration molecular weight markers) were also run on the same column after two-three bed volume washings of the column in the same buffer (0.02 M sodium phosphate buffer (pH 7.8). The molecular weights of different peaks in the chromatogram of TPP treated alpha chymotrypsin were calculated from the plot of log molecular weight vs retention volume of the molecular weight markers.

### Transmission electron microscopy

Transmission electron micrographs were recorded on a Philips CM-10 instrument equipped with digital imaging at AIIMS, New Delhi. A drop of protein solution from each alpha chymotrypsin preparation was placed on a copper grid and dried.

### Scanning electron microscopy

Scanning electron micrographs of each alpha chymotrypsin samples were observed by using a Carl Zeiss EVO5O scanning electron microscope (SEM) instrument at IIT Delhi. Samples were mounted on a circular metallic sample holder with a sticky carbon tape.

## Supporting Information

Figure S1 **SDS-PAGE of native alpha chymotrypsin.** Lane 1: molecular weight markers; Lane 2: Alpha chymotrypsin (20 µg); Lane 3: Alpha chymotrypsin (60 µg). SDS-PAGE was performed under non-reducing conditions (without β-mercaptoethanol and without any boiling of the sample). The sample buffer contained 0.5 M Tris-HCl buffer, pH 6.8; 10% SDS; 0.5% Bromophenol Blue and 50% glycerol.
(TIF)

Figure S2 **NTA Video showing the Brownian motion of native alpha chymotrypsin.**
(WMV)

Figure S3 **NTA Video showing the Brownian motion of TPP treated alpha chymotrypsin (55% IP).** Alpha chymotrypsin preparation obtained by three phase partitioning using 55% saturation of ammonium sulphate.
(WMV)

Figure S4 **NTA Video showing the Brownian motion of TPP treated alpha chymotrypsin (65% IP).** Alpha chymotrypsin preparation obtained by three phase partitioning using 65% saturation of ammonium sulphate.
(WMV)

Figure S5 **NTA Video showing the Brownian motion of TPP treated alpha chymotrypsin (75% IP).** Alpha chymotrypsin preparation obtained by three phase partitioning using 75% saturation of ammonium sulphate.
(WMV)

Figure S6 **HPLC gel filtration chromatograms on TSK G2000SWXL column of major peaks** (Figure S6A-Peak2 (Alpha-C); Figure S6B-Peak 1a; Figure S6C-Peak 1b; Figure S6D-Peak 1c) of alpha chymotrypsin preparations after gel filtration chromatography on Sephadex G-200.
(TIF)

Figure S7 **Calibration Plot using Gel filtration molecular weight markers.** Inlay shows the calculated molecular mass of the fractions (peak fractions obtained from the chromatogram of Figure 5) using the above calibration plot.
(TIF)

Figure S8 **SEM images of various preparations of TPP treated alpha chymotrypsin.** (A) Alpha-C (B) 55% IP (C) 65% IP (D) 75% IP.
(TIF)

Figure S9 **TEM images of various preparations of TPP treated alpha chymotrypsin.** (A) Alpha-C (B) 55% IP (C) 65% IP (D) 75% IP.
(TIF)

Figure S10 **Surface hydrophobicity measurement of various preparations of TPP treated alpha chymotrypsin by ANS titration using Fluorescence spectroscopy.** (A) Alpha-C (B) 50% IP (C) 55% IP (D) 80% IP. Slope of the regression line shows the surface hydrophobicity index (S_o_).
(TIF)
