# Supplementary figures and images for: Activation of Alpha Chymotrypsin by Three Phase Partitioning Is Accompanied by Aggregation
Source: PLoS One. 2012 Dec 11;7(12):e49241. doi: 10.1371/journal.pone.0049241 (PMC3519768; doi:10.1371/journal.pone.0049241)

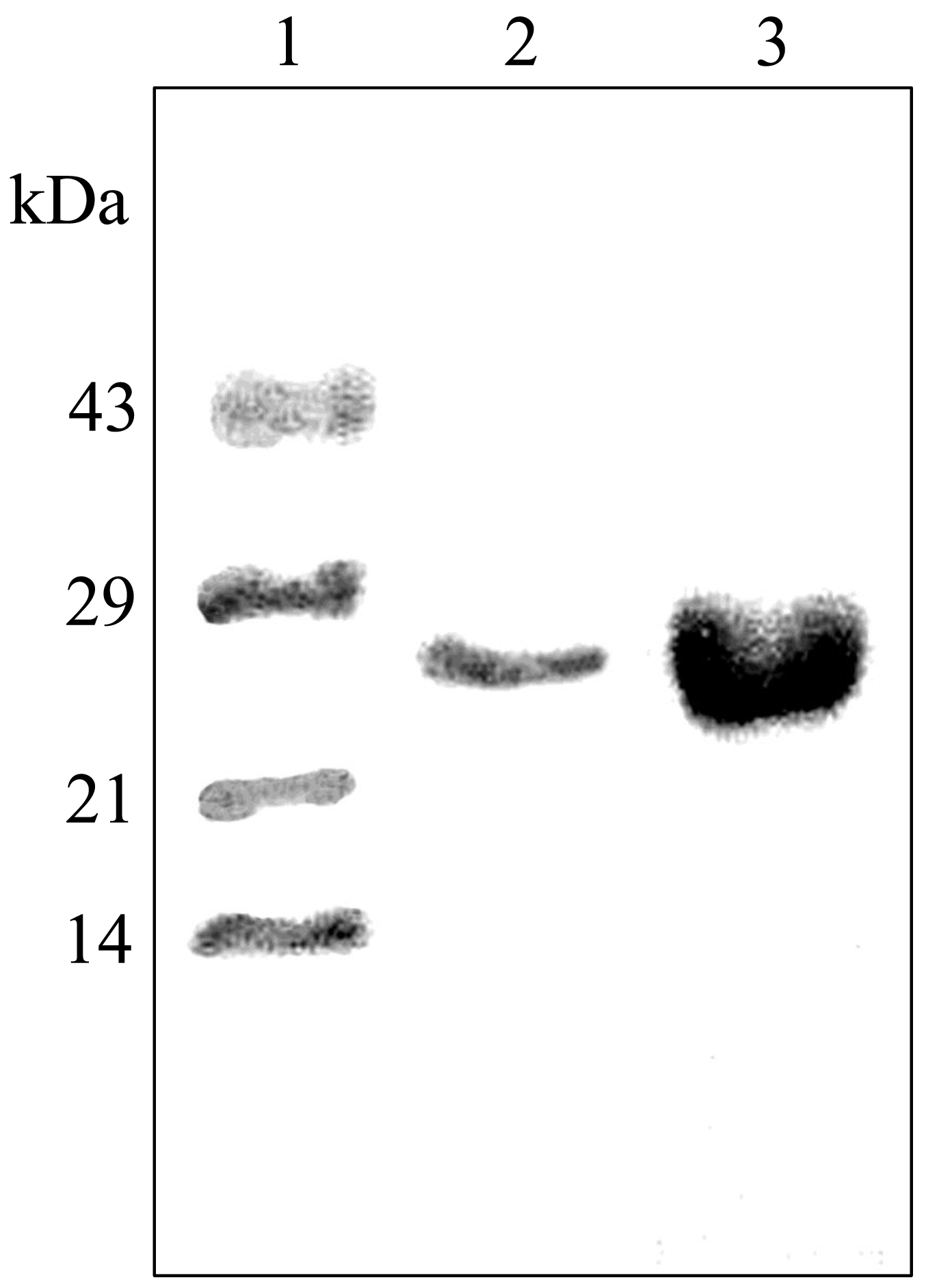

Supplement: Figure S1 — SDS-PAGE of native alpha chymotrypsin. Lane 1: molecular weight markers; Lane 2: Alpha chymotrypsin (20 µg); Lane 3: Alpha chymotrypsin (60 µg). SDS-PAGE was performed under non-reducing conditions (without β-mercaptoethanol and without any boiling of the sample). The sample buffer contained 0.5 M Tris-HCl buffer, pH 6.8; 10% SDS; 0.5% Bromophenol Blue and 50% glycerol. (TIF) [file pone.0049241.s001.tif]

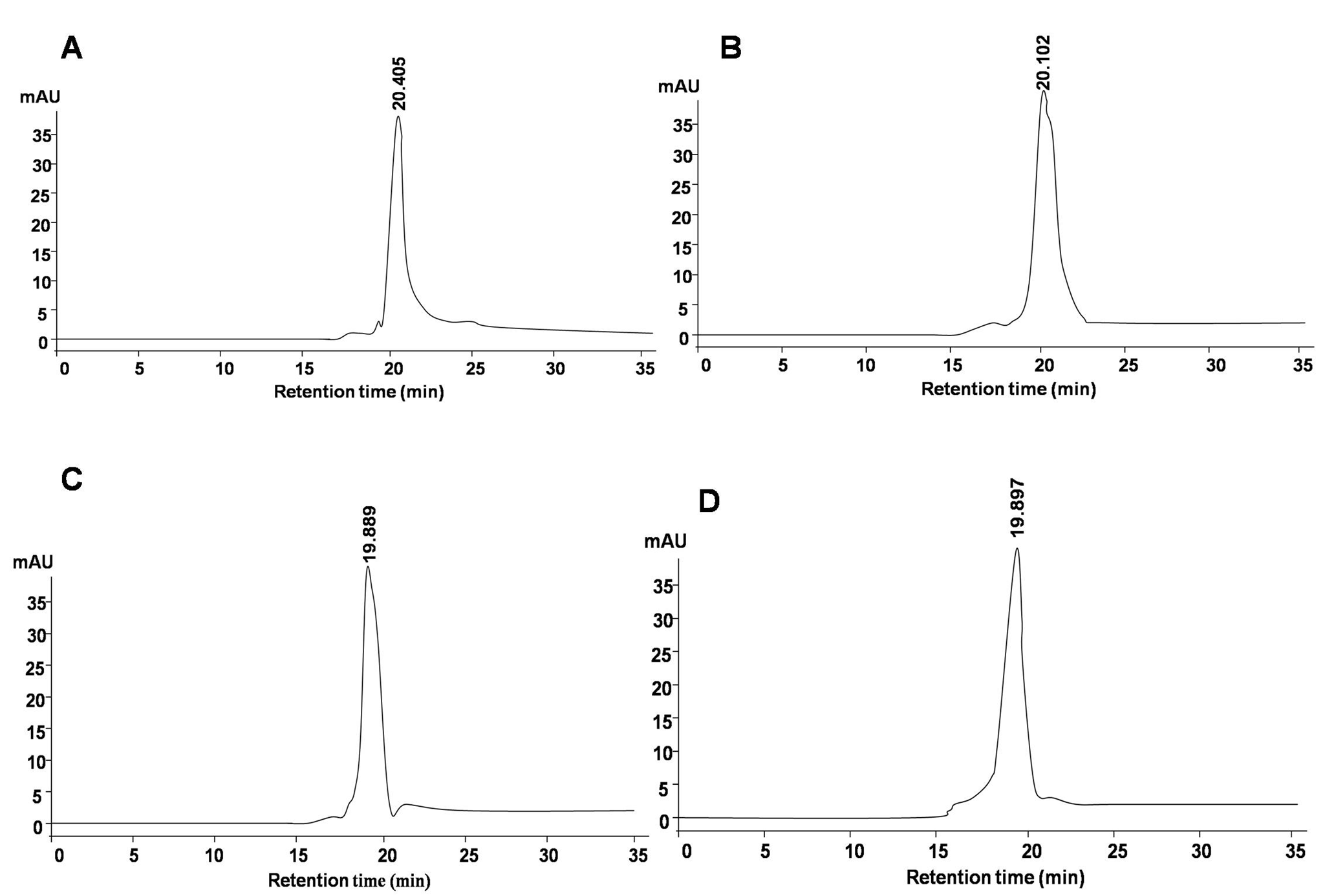

Supplement: Figure S6 — HPLC gel filtration chromatograms on TSK G2000SWXL column of major peaks (Figure S6A-Peak2 (Alpha-C); Figure S6B-Peak 1a; Figure S6C-Peak 1b; Figure S6D-Peak 1c) of alpha chymotrypsin preparations after gel filtration chromatography on Sephadex G-200. (TIF) [file pone.0049241.s006.tif]

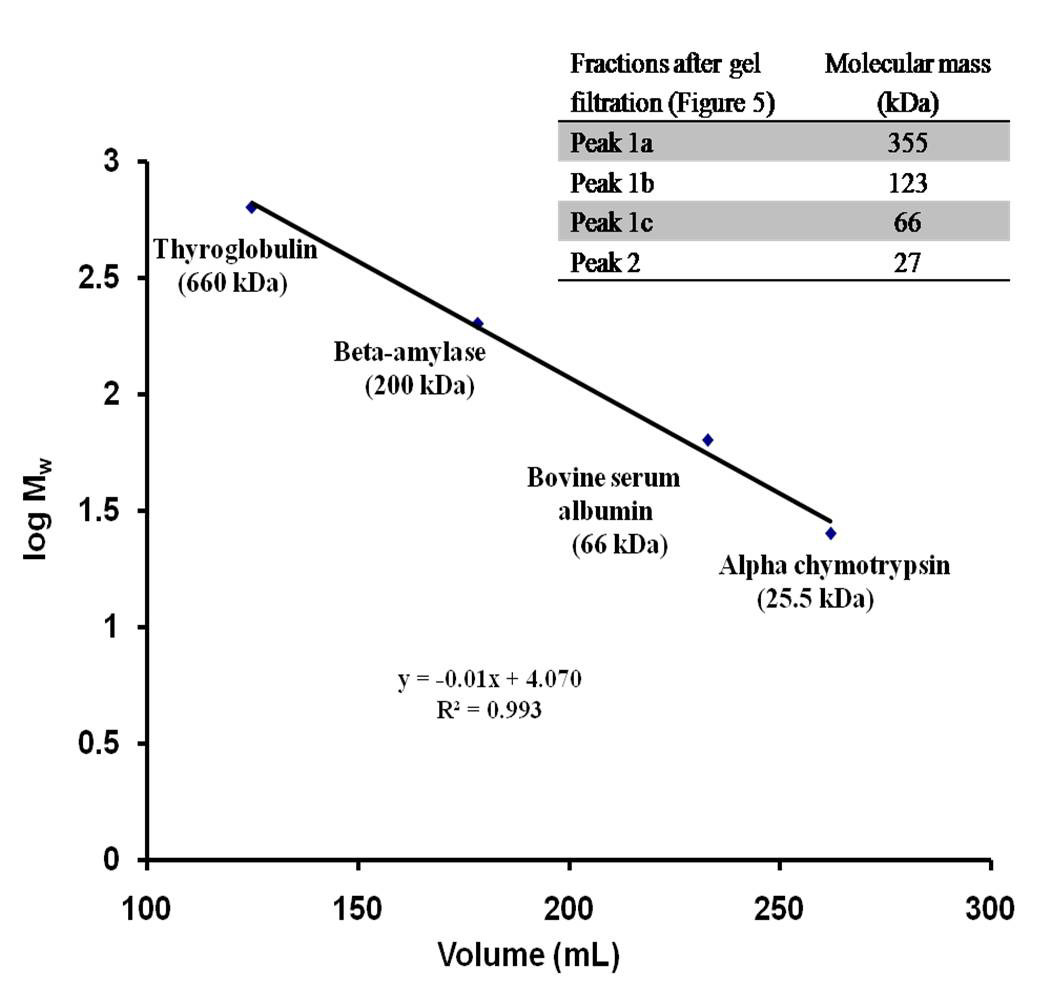

Supplement: Figure S7 — Calibration Plot using Gel filtration molecular weight markers. Inlay shows the calculated molecular mass of the fractions (peak fractions obtained from the chromatogram of Figure 5) using the above calibration plot. (TIF) [file pone.0049241.s007.tif]

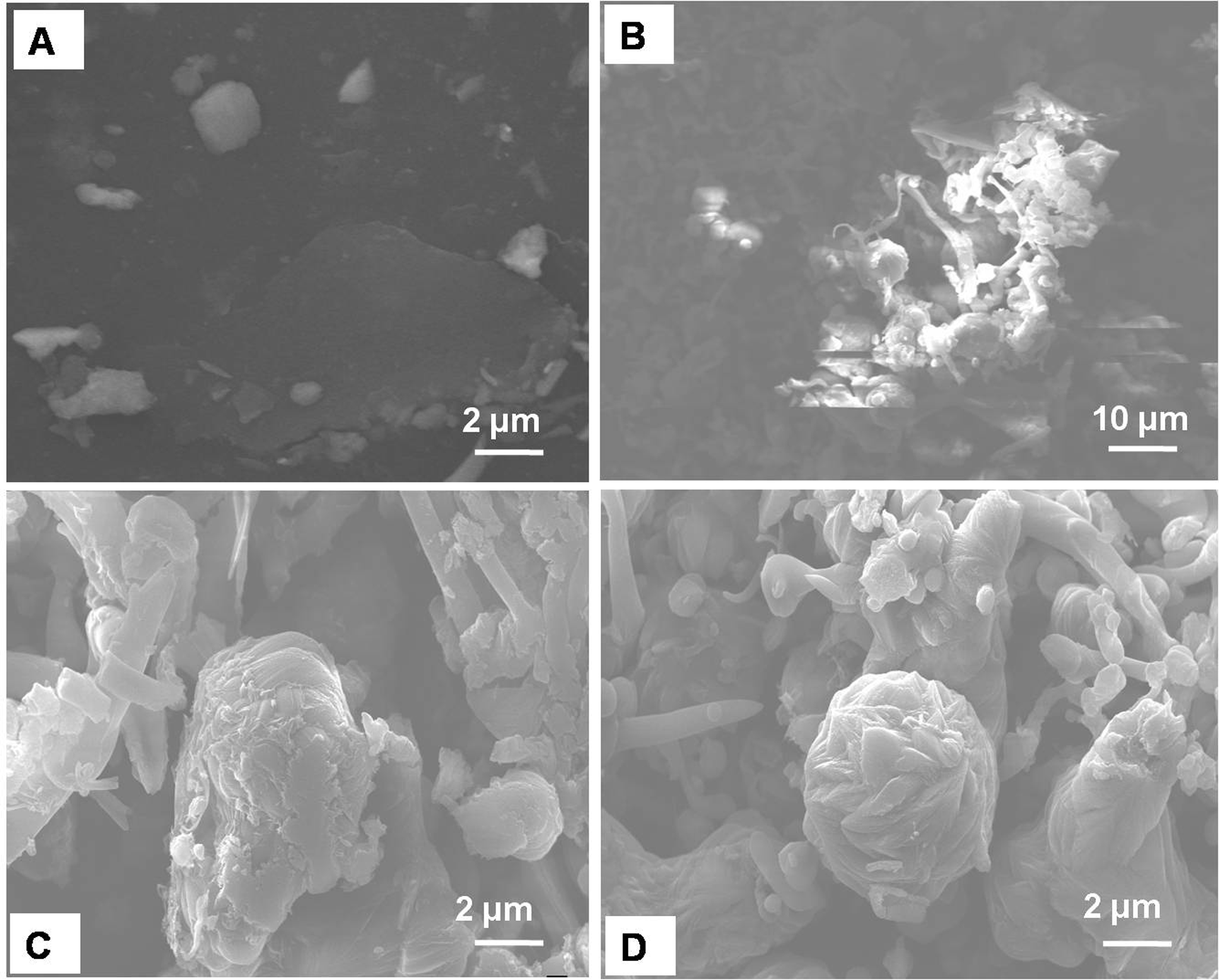

Supplement: Figure S8 — SEM images of various preparations of TPP treated alpha chymotrypsin. (A) Alpha-C (B) 55% IP (C) 65% IP (D) 75% IP. (TIF) [file pone.0049241.s008.tif]

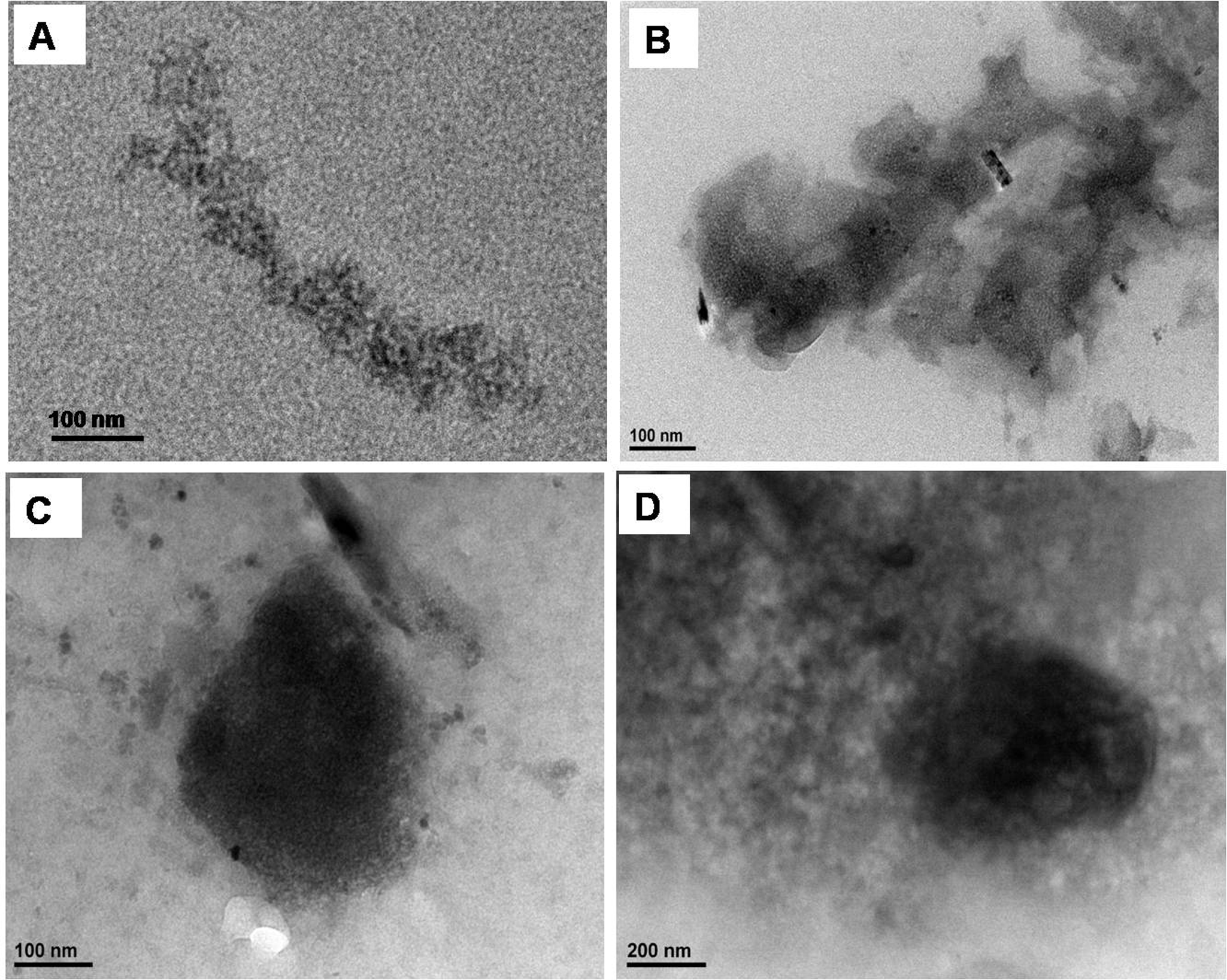

Supplement: Figure S9 — TEM images of various preparations of TPP treated alpha chymotrypsin. (A) Alpha-C (B) 55% IP (C) 65% IP (D) 75% IP. (TIF) [file pone.0049241.s009.tif]

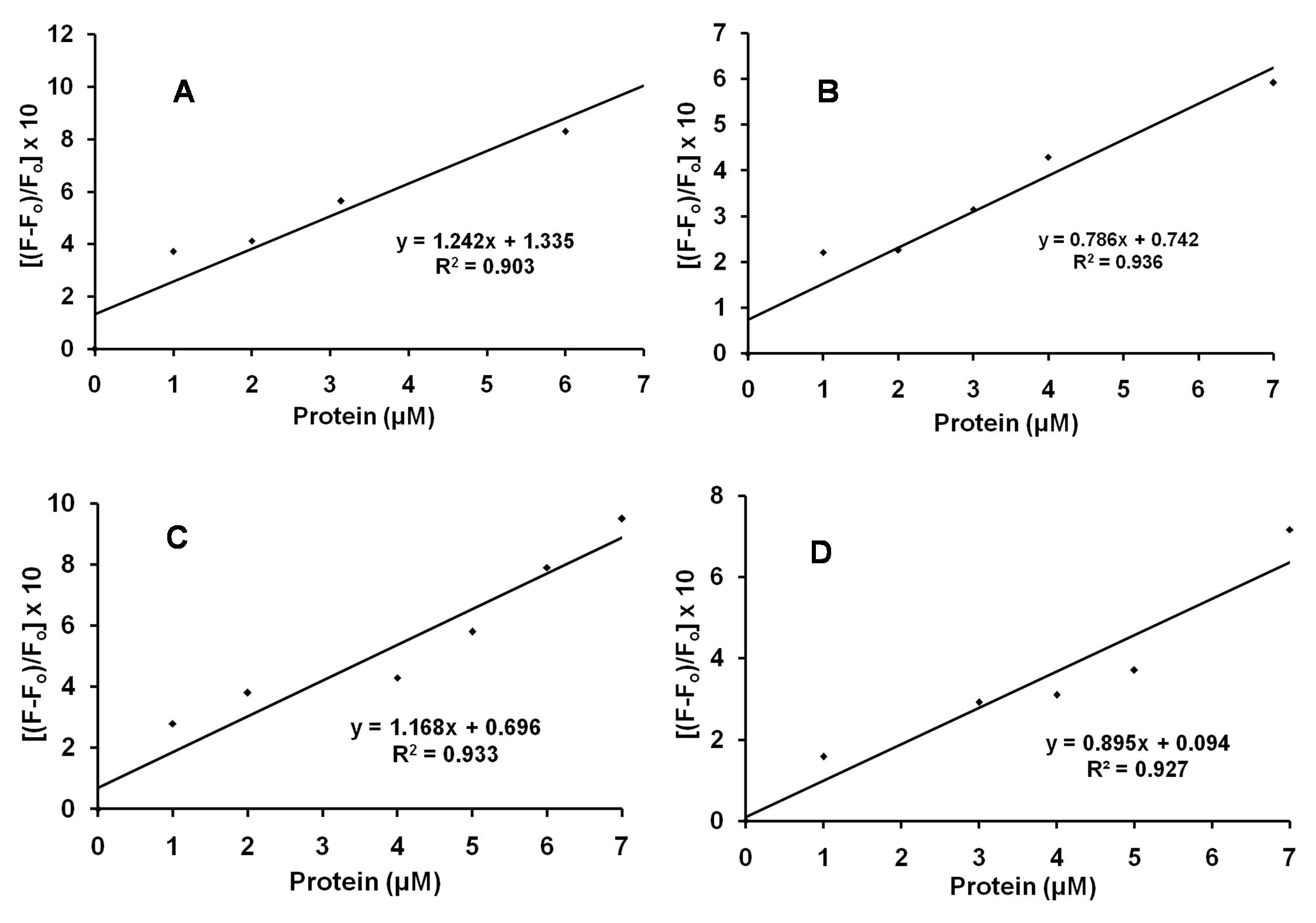

Supplement: Figure S10 — Surface hydrophobicity measurement of various preparations of TPP treated alpha chymotrypsin by ANS titration using Fluorescence spectroscopy. (A) Alpha-C (B) 50% IP (C) 55% IP (D) 80% IP. Slope of the regression line shows the surface hydrophobicity index (So). (TIF) [file pone.0049241.s010.tif]
